# Ischemic and non-ischemic myocardial injuries at autopsy- an overview for forensic pathologists

**DOI:** 10.1007/s00414-025-03479-1

**Published:** 2025-04-02

**Authors:** Katarzyna Michaud, Cristina Basso, Hans H. de Boer, Tony Fracasso, Monica de Gaspari, Carla Giordano, Xiaofei Li, Joaquin Lucena, Pilar Molina, Sarah Parsons, Mary N. Sheppard, Allard C. van der Wal, On behalf of the Association for European Cardiovascular Pathology

**Affiliations:** 1 University Center of Legal Medicine Lausanne - Geneva, Lausanne University Hospital and University of Lausanne, Lausanne, Switzerland; 2 Cardiovascular Pathology Unit, Department of Cardiac, Thoracic and Vascular Sciences and Public Health, University of Padua, Padua, Italy; 3 Department of Forensic Medicine, Victorian Institute of Forensic Medicine, Monash University, Southbank, VIC Australia; 4 Geneva University Hospital and University of Geneva, Geneva, Switzerland; 5 Department of Radiological, Oncological and Pathological Sciences, Sapienza University of Rome, Rome, Italy; 6 Department of Pathology, Maastricht University Medical Center (MUMC), Maastricht, The Netherlands; 7 Department of Pathology Institute of Legal Medicine and Forensic Sciences, Seville, Spain; 8 Department of Pathology, Institute of Legal Medicine and Forensic Sciences, Valencia, Spain; 9 Research group CAFAMUSME, La Fe Health Research Institute, Valencia, Spain; 10 CRY Cardiovascular Pathology Unit, Cardiovascular and Genetic Research Institute, City St George’s, University of London, London, UK

**Keywords:** Myocardial infarction, Myocardial injuries, Autopsy, Forensic autopsy, Myocarditis, Cocaine, Catecholamines, Toxicology, Reperfusion injury, Violent death, Natural death

## Abstract

Cardiovascular diseases are major causes of morbidity and death worldwide, and most cardiac deaths are related to ischemic injury of the myocardium (myocardial infarction). As underlined in the current clinical definition and classification of myocardial infarctions, not all myocardial injuries are due to ischemia: irreversible injury, ending in necrosis, can be induced also by various other factors, such as infections, immune disorders, physical and chemical agents, and trauma. This is supported by clinical studies showing that elevated serum levels of cardiac troponins, as a measure of myocardial damage, are also a common finding in the non-ischemic types of myocardial injury. Forensic pathologists confronted with autopsy findings suggestive of myocardial injury should therefore realize that both ischemic and non-ischemic forms of myocardial death can be observed, and not only in natural but also non-natural deaths (intoxications, asphyxia, traumatic and iatrogenic deaths, and others). Distinguishing these different types of injuries and underlying diseases or circumstances of death is critical, not only to determine the cause and mechanism of death, but also to help investigate often challenging medico-legal scenarios. This article reviews the broad spectrum of ischemic and non-ischemic myocardial injuries in natural and violent deaths. From this perspective we propose a diagnostic approach to myocardial injuries in a forensic pathology context.

## Key points

- Cell injury is a well-known term in pathology but can be confusing for forensic pathologists since it alludes to both natural and non-natural circumstances of cell death.

- Not all myocardial injuries are ischemic. A discrimination should made between ischemic injury (infarction) and non-ischemic injuries (for example myocarditis).

- Myocardial infarction type 2 and several forms of non-ischemic myocardial injuries can be the consequence of natural but also violent causes of death.

- Ancillary techniques (postmortem imaging, immunohistochemistry (IHC), NBT stain and postmortem chemistry) allow to detect myocardial injuries, but they cannot discriminate between ischemic and nonischemic causes of cell death. IHC can be a useful tool to delineate even very small foci of myocardial injuries in the heart.

## Introduction

The examination of the heart is a crucial part of both clinical and forensic autopsy. Myocardial injury is a common finding at autopsy, given the wide variety of diseases and pathophysiological processes that can affect the myocardium, together with the relative high susceptibility of cardiomyocytes to irreversible cell damage. Interpretation of myocardial injury at autopsy can be challenging. Cardiac pathologies such as thrombosis superimposed on coronary atherosclerosis, established myocardial infarction, fulminant myocarditis, complex congenital heart disease or a well-defined primary cardiomyopathy may readily explain the cause of death. However, other findings may be less clear, such as subtle forms of cardiomyopathy where the heart can be macroscopically normal, in cases of insignificant coronary atheroma or when the causes of myocardial damage are extracardiac.

The most common cause of cardiomyocyte injury is ischemia [1]. In such cases, critical flow reduction in a coronary artery leads to ischemic myocardial injury in the perfusion territory of the artery, and, if persistent, progresses to myocardial infarction. However, besides ischemia, myocardium can also be damaged by various other types of injuries such as infections, immune mediated diseases, genetically determined cardiomyocyte damage, physical and chemical agents, and trauma. Forensic pathologists frequently encounter clinical information and/or autopsy findings indicative of myocardial injury. Discriminating between the various ischemic and non-ischemic causes is of vital importance for determining cause and mechanism of death, but also aids in investigating often-challenging medico-legal scenarios. Such scenarios include, motor vehicle incidents potentially caused by sudden cardiac arrest, homicide cases that also have severe underlying cardiac disease, post-surgical deaths, and myocardial injury in the setting of cardio-pulmonary resuscitation (CPR) and others. Various violent insults, especially when there is a long agonal period, can be a cause of myocardial injury through the excessive catecholamines release. If these injuries are confused with ischemia/infarction, death may be mistakenly assumed to be a natural process. This article reviews the pathophysiology and post-mortem diagnosis of myocardial injuries that are most relevant to routine general and forensic autopsy practice.

### Myocardial injury: definitions and cellular pathways

It should be noted that myocardial injury involves not only the cardiomyocytes but also the interstitium and intramyocardial vessels. Damage to these different components may underly also distinct types of pathology. Myocyte injury, which is associated with many acquired myocardial pathologies, occurs when external stresses to cardiomyocytes exceeds their adaptive capacities [2]. Such injury can result from hypoxia/ anoxia, drugs, trauma, radiation, immunological effects, and infectious diseases.

Myocardial injury can be to some level reversible but if the stress is severe and/or persistent, injured cells pass a nebulous “point of no return”, it progresses to irreversible cell damage and cell death. Sublethal injuries can manifest as reversible states ofmyocardial injury but can on the longer term also evolve to death/disappearance of cells. Myocyte injury is characterized by intracellular accumulation of fluid and lysis of myofibrils, but the cells still retain their nuclei. This descriptive histological pattern is often referred to as “*myocytolysis”* and has been reported in various pathological conditions which include, myocardial ischemia, intoxications, myocarditis, storage disease and others. There are several pathways of cell injury that may lead to irreversible injury of myocytes and cell death. These pathways can be unregulated like oncosis and necrosis or regulated and programmed like apoptosis, necroptosis and pyroptosis. *Oncosis* is a process of cell injury with cell swelling and karyolysis and is a precursor of unregulated cell death leading to necrosis. *Apoptosis* is a form of regulated or programmed cell death with cell shrinkage, pyknosis and karyorrhexis. *Necroptosis* shows hybrid features of apoptosis and oncosis. *Pyroptosis* occurs in cells infected by microorganisms. In case of ischemic injury, which underlies myocardial infarctions, initial oncosis followed by necroptosis and finally necrosis represents the dominant pathway of cardiomyocyte injury and death, whereas apoptosis has only a secondary role involving mostly non myocytic components such as interstitial cells and microvasculature. In myocardial infarctions its contribution to the final loss of myocardium is considered much less compared to necrosis [3].

*Necrosis* is mediated by a failure of the cell’s energy metabolism and with disruption of cell membrane and can be considered a degenerative process that occurs after cell death. Its characterized histologically by increased eosinophilia, cytoplasmic homogenization and nuclear pyknosis (chromatin condensing), followed by disappearance of the nucleus in one to two days. Morphologic appearance of necrosis can be classified histologically as coagulative, liquefactive, gangrenous, caseous, fat or fibrinoid [2]. Irreversible ischemic damage is the hallmark of myocardial infarction, and results in ischemic necrosis, which is histologically characterized by a *coagulative type of necrosis* of cardiomyocytes (opaque eosinophilic cytoplasm and disappearance of nuclei).

*Contraction band necrosis* (CBN) of cardiomyocytes is another form of acute myocardial injury, which arises approximatively 10 min after onset of acute injury and is characterized by the formation of thick eosinophilic bands in the cytoplasm of cardiomyocytes due to clustering of hypercontracted contractile proteins. These histological features relate to a high permeability of damaged myocytes leading to massive influx of Ca2 + ions followed by irreversible hypercontraction. CBN may be a feature of coronary reperfusion after the onset of ischemic injury but can also occur in situations of nonischemic myocardial injury. Both as a result of a complication of a natural disease, but also due to accidental or violent causes. They are discussed in more detail in the following chapters. In the ancient literature, irreversible contraction is described as “tetanic death” (cardiomyocytes are stuck in irreversible contraction) [4].

*Autophagy* is another cellular process which involves the segregation of damaged organelles, aged proteins, and other intracellular contents into ‘autophagic vacuoles’, followed by further clearance in order to maintain intracellular homeostasis. Autophagy can increase the severity of injury, as occurs in circumstances where inflammatory responses are strongly triggered (such as ischemia followed by reperfusion) [5]. However, in other scenarios such as in chronic forms of ischemia (hibernation, see later) it may contribute to myocyte survival [3, 6].

The modes of cell death as mentioned above, which are obviously of interest from a pathophysiological point of view, are difficult or even impossible to identify in histological sections at autopsy, and often require advanced laboratory techniques for investigation. From a practical point of view, only necrosis, myocytolysis (see below) and CBN can usually be easily recognized histologically in the autopsied heart.

**Fig. 1:**
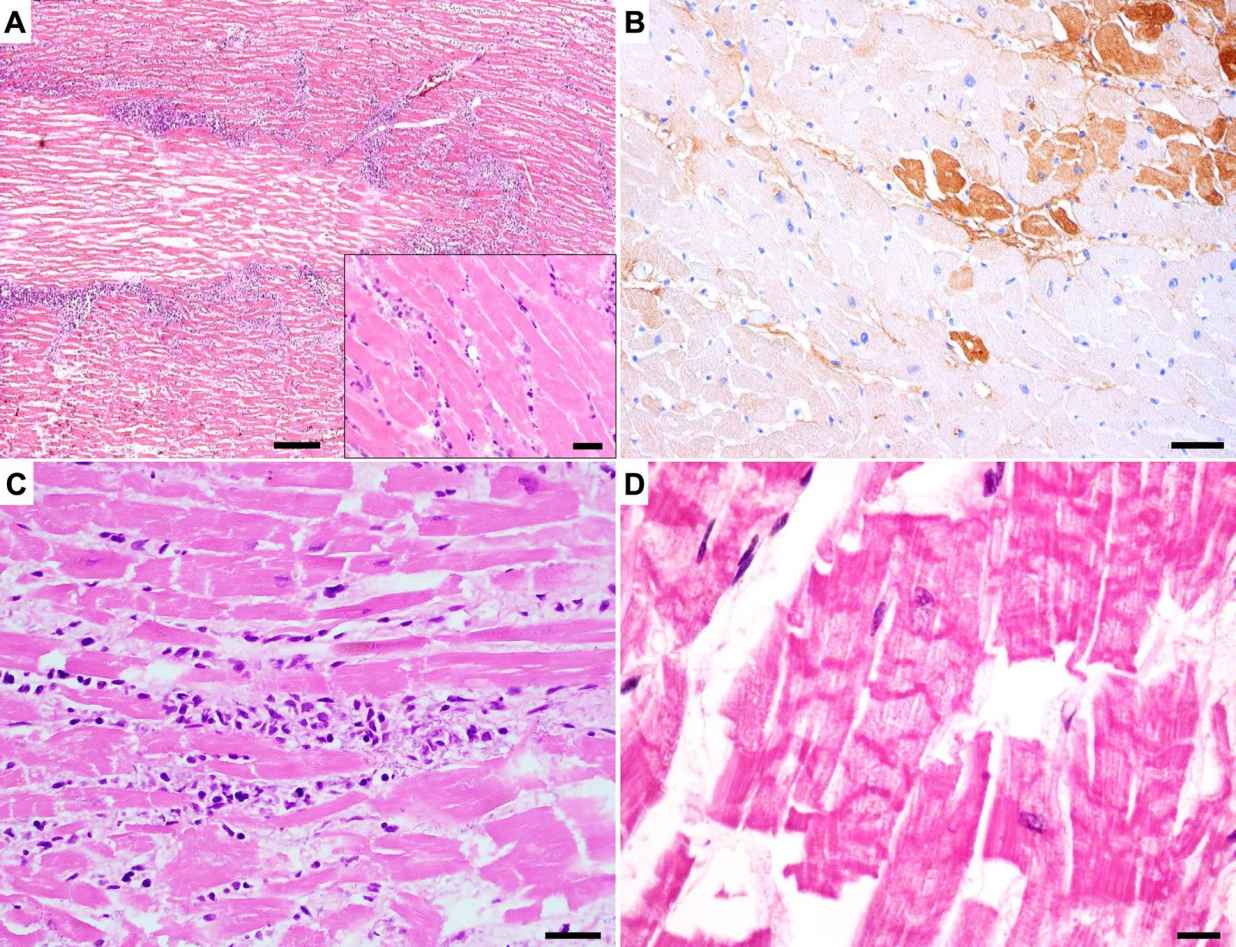
Histology of various types of acute myocardial injury. **A**) Overview of the border-zone of a myocardial infarction one week after onset of ischemic injury. Insert shows detail of coagulative necrosis, H&E stain, bar = 100 μm, bar in the box 30 μm. **B**) Microscopic infarctions one day after onset of ischemic injury showing clusters and isolated necrotic cells, immune-stained with anti-fibrinogen antibody (brown), bar = 30 μm. **C**) Acute myocytolysis in viral myocarditis, showing cardiomyocyte damage by mononuclear inflammatory cells, H&E stain, bar = 30 μm. **D**) Contraction band necrosis of myocytes, H&E stain, bar = 10 μm

Various forms of acute myocardial injuries are illustrated in Fig. 1.

#### Chronic and healed myocardial injuries

*Colliquative myocytolysis* (which should be distinguished from the forms of acute myocytolysis as described above) occurs in prolonged situations of non-lethal myocyte injury, which causes chronic depletion of energy in the cell, resulting in a decrease of cellular functions including impaired proteins synthesis. Histologically, colliquative myocytolysis shows a large cytoplasmic vacuole surrounded by a thin eosinophilic rim due to the reduction of cytoplasmic myofibers. It can be observed in severely dilated hearts of any type (ischemic and non-ischemic). In case of chronic ischemic injury, it can be noticed at the periphery of myocardial infarcts, and in the subendocardial and perivascular regions. Also, the so-called “hibernating” areas of myocardium, a form of sublethal injured dysfunctional myocardium due to longstanding hypoxic/ischemic injury, reveals prominent colliquative myocytolysis, and can easily be recognized [7–12]. In the literature, “colliquative myocytolysis” is often described as simply “myocytolysis” which may lead to confusion, and should therefore be strictly reserved for injured myocytes with large cytoplasmic vacuoles [7].

In response to injury, the presence of necrosis evokes an inflammatory response, injured myocytes are engulfed by granulocytes (PMN), and/or lymphocytes and macrophages (depending on the type of inciting agents), followed by stages of repair, initially a fibrocellular granulating tissue reaction and finally ending up in *fibrosis* (scar tissue). Scars stabilize the vulnerable injured lesion. There is no (significant) regeneration at the sites of loss of viable myocardium, which is compensated by hypertrophy of the adjacent vital ventricular myocardium (compensatory hypertrophy). However, significant loss of myocardium (such as in myocardial infarctions) will lead to congestive heart failure and arrhythmias, up to cardiogenic shock. Early stages of myocardial injuries may induce electrical instability in the heart leading to ventricular arrhythmias up to ventricular fibrillation (VT), as has been described in detail for ischemic injuries / myocardial infarctions. But also postinfarction fibrotic scars containing interspersed strands of viable of cardiomyocytes may serve as a site for the generation re-entry arrhythmias which eventually can end up in ventricular fibrillation (VF) [13, 14]. These sequential steps of healing may occur in the setting of ischemic and non-ischemic injuries.

**Fig. 2:**
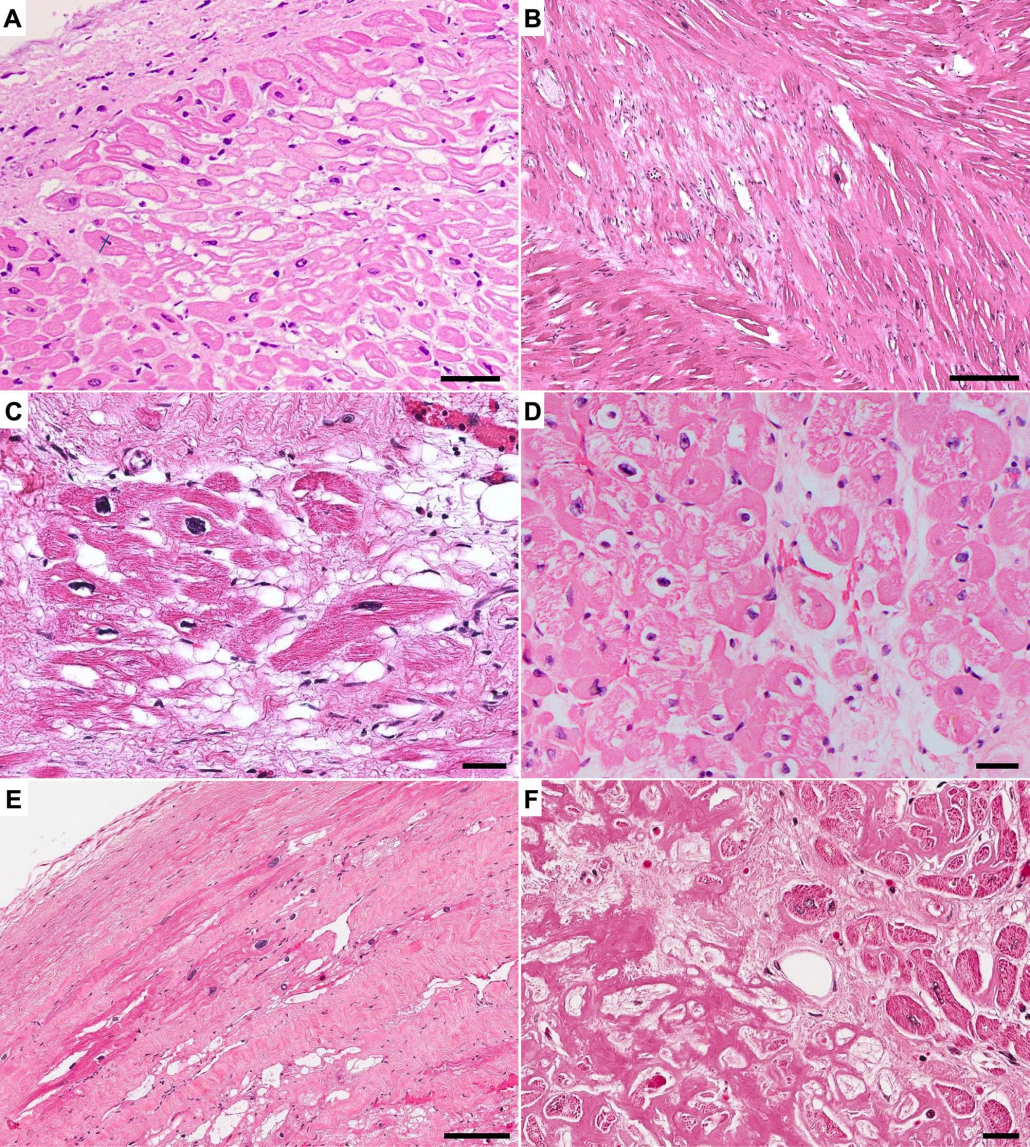
Histology of chronic myocardial injury: colliquative myocytolysis, all are H&E stains. **A**) Subendocardial chronic injury in case of ischemic heart failure (left ventricle). bar = 100 μm. **B**) Hypertrophic cardiomyopathy (HCM): myocytolysis coupled with myocyte disarray, bar = 200 μm. **C**) Arrhythmogenic cardiomyopathy (ACM): myocytolysis in association with fibro-fatty replacement of myocardium, bar = 30 μm. **D**) Dilated cardiomyopathy (DCM): myocytolysis with large perinuclear haloes, bar = 30 μm. **E**) Chronic ischemic heart disease: subendocardial dense scar with interspersed vacuolated myocytes, bar = 50 μm. **F**) Amyloidosis: interstitial deposition of eosinophilic, amorphous and homogeneous material and chronic myocyte injury with cytoplasmic attenuation/atrophy and vacuolization, bar = 30 μm

Chronic and healed myocardial injuries are illustrated in Fig. 2.

### Causes of myocardial injuries

*Myocardial injury* encompasses any form of myocardial damage or destruction. In clinical practice, the use of high-sensitivity troponin (Hs Tn) assays has revealed that irreversible forms of myocardial injury leading to necrosis are common finding in a wide range of clinical presentations. While substantially elevated Hs Tn levels have a high predictive value (over 90%) for acute myocardial infarctions due to coronary atherothrombosis, elevations up to three times the upper reference limit are less specific and may be associated with other ischemic processes, or even non-ischemic conditions (“non-ischemic injuries”) (Tables 1 and 2). Moreover, ischemic and non-ischemic pathways of myocardial injury can co-exist, further complicating diagnosis.

**Table 1:** Ischemic injuries in cases of natural and non-natural manner of death (MOD)

MOD	Causes of death	Comments
**Natural**	Coronary atherothrombosis	• Erosion or rupture of coronary atherosclerotic plaque leading to superimposed thrombus formation with partial (mural) or total occlusion of the vascular lumen (MI type 1)
	Fixed coronary atherosclerotic plaques (> 75% stenosis)	• Significant coronary plaque stenosis (without thrombus) in combination with LV dilatation and/ or non-coronary causes of oxygen demand-supply imbalance (MI type 2)
	Other cardiac causes of oxygen demand-supply	• Non-atherosclerotic coronary artery diseases (spasm/small vessel diseases / dysfunction, vasculitis, dissection, embolism, congenital anomalies, fibromuscular dysplasia) (MI type 2) • Non-coronary cardiac causes of oxygen demand-supply imbalance (sustained tachyarrhythmias, brady-arrhythmias, LV hypertrophy and/or dilatation), severe hypertension (MI type 2)
	Extra-cardiac oxygen demand- supply disbalance	• Severe anemia, respiratory failure, physical exertion especially in combination with fixed coronary atherosclerotic plaques (MI type 2)
**Non-natural**	Extra-cardiac oxygen demand- supply disbalance (MI type 2)	• Hypovolemic shock (external/internal hemorrhage) • Oxygen demand-supply imbalance after non-cardiac surgery • Hemodynamic stress/hypoperfusion in cases with prolonged coma (long agony), in the setting of asphyxia, carbon monoxide poisoning, drugs intoxications etc.
	Cocaine intoxication	• Coronary spasm, atherosclerosis, thrombosis, etc. (MI type 1 or/and MI type 2)
	Coronary procedural events related pathology	• PCI and CABG related ischemia including stent or graft thrombosis (MI type 4 and 5), coronary dissection, embolism, reperfusion injuries etc.
	Cardiac allograft failure	• Chronic allograft vasculopathy (epicardial and small vessels disease) (MI type 1 or/and MI type 2)

**Table 2:** Non-ischemic myocardial injuries in cases of natural and non-natural manner of death (MOD)

MOD	Causes	Comments
**Natural**	Infections Immune disorders	• Myocarditis (viral, bacterial, fungi)
	Structural heart disease (including cardiomyopathies)	• Chronic forms of injury: adrenergic, RAAS, cytokine / inflammation and mechanical stresses
	Chronic kidney disease	• Toxic uremic effects and hemodynamic stress
	Takotsubo syndrome, neurocardiogenic injury, pheochromocytoma	• Catecholamine induced myocardial injuries
	Sepsis	• Massive release of cytokine, catecholamine induced injuries, etc.
**Non-natural**	Therapeutic (as cancer therapy) and illicit drugs (as cocaine), vaccination	• Toxic myocardial injuries/ myocarditis/ cardiomyopathies • Hypersensitivity myocardial injuries • Catecholamine induced myocardial injuries
	Radiation	• Various mechanisms of myocardial cell death
	Blunt trauma to the heart	• Cardiac contusion or rupture
	Traumatic brain injuries	• Catecholamine induced myocardial injuries
	CPR with short survival on intensive care	• Traumatic and reperfusion injuries
	Extremal physical and emotional stress including a third person involvement or external event	• Catecholamine induced myocardial injuries
	Cardiac surgery, TAVI and ablation procedures, stent implantation	• Ischemic, non-ischemic and reperfusion myocardial injuries
	Cardiac allograft failure	• Immune injury to cardiomyocytes (myocarditis) and microvessels: cellular and humoral rejection

### Myocardial injury due to ischemia

#### Pathology

Acute ischemic injury is usually caused by critical coronary flow insufficiency. The high oxygen demands of cardiomyocytes will lead to almost immediate functional impairment. “Very early” (reversible) ischemic changes are characterized by mitochondrial swelling and sarcolemmal disruptions in cardiomyocytes and can be observed only at ultrastructural examination with transmission electron microscopy. The period of reversible injury lasts for approximately 15 min and if ischemic insult persists, it is followed by irreversible damage within 20–60 min. Morphologically, the earliest histological signs of irreversible relaxation (“atonic death”) are characterized by regional thinning of the wall with a wavy pattern of cardiomyocyte bundles, hypereosinophilia and interstitial oedema. However, these changes may have poor reliability in histological evaluations and depend on autopsy interval and resuscitation and thus may be subject to over-interpretation [15]. The time of onset of myocardial infarction depends on situations such as severity of coronary occlusion (total or partially), presence of collaterals in the myocardial area at risk (protective), or eventually preconditioning (resistance/protection to ischemia due to previous ischemic episodes). Leakage of intracellular proteins at the time of cell membrane disruptions provides a means of detecting tissue-specific necrosis using blood or serum samples with raised troponins, and other cardiomyocyte proteins (LDH, CPK etc.) in the serum of patients with acute coronary syndromes (and other types of injury).

#### Clinical backgrounds

A clinical diagnosis of myocardial infarction is based on the presence of elevated cardiac troponin levels, in combination with prolonged chest pain, ECG recordings or regional wall motion abnormalities on imaging/severe narrowing and/or angiographic detection of a coronary thrombus. The most recent, 4th universal definition of myocardial infarction, as formulated and described in detail by four international cardiac societies (ESC, AHA, WHF and ACC) [1] distinguishes 5 types of myocardial infarction.

Of particular clinical and pathological interest, is the distinction between the types 1 and 2 infarctions. *Type 1 MI* (T1MI) refers to MI that result from acute coronary artery atherothrombosis, be it mural or totally occlusive. These are encountered in circa 80% of patients with acute coronary syndromes (ACS). *Type 2 MI* is not due to acute coronary plaque disruption and thrombosis but relate to other causes that may cause or at least contribute to oxygen supply-demand imbalance leading to myocardial injury (see Table 1). The various underlying causes include cardiac and extra-cardiac diseases or processes. Reported frequencies of type 2 MI vary from 10 to 30% of all MI patients.

*Type 3* represents a group of patients who witness a sudden arrhythmic following a short period of clinical ischemic symptoms, but without evidence of injury (no elevation of cardiac troponins). However, in a proportion of these victims atherothrombotic occlusion can be found at autopsy, stressing the importance of carefully slicing the coronary arteries [16].

*Type 4 and 5* are myocardial infarctions that occur in a setting of percutaneous coronary interventions (PCI, including stent implantation) or coronary artery bypass graft (CABG) surgery. Both procedures can be associated with fatal complications and therefore are relevant for autopsy practice. Complications include but are not limited to acute or late stent thrombosis, coronary artery ruptures or dissection, embolization by guidewire coating material etc., which could have medicolegal consequences [15].

### Myocardial ischemia and reperfusion

**Fig. 3:**
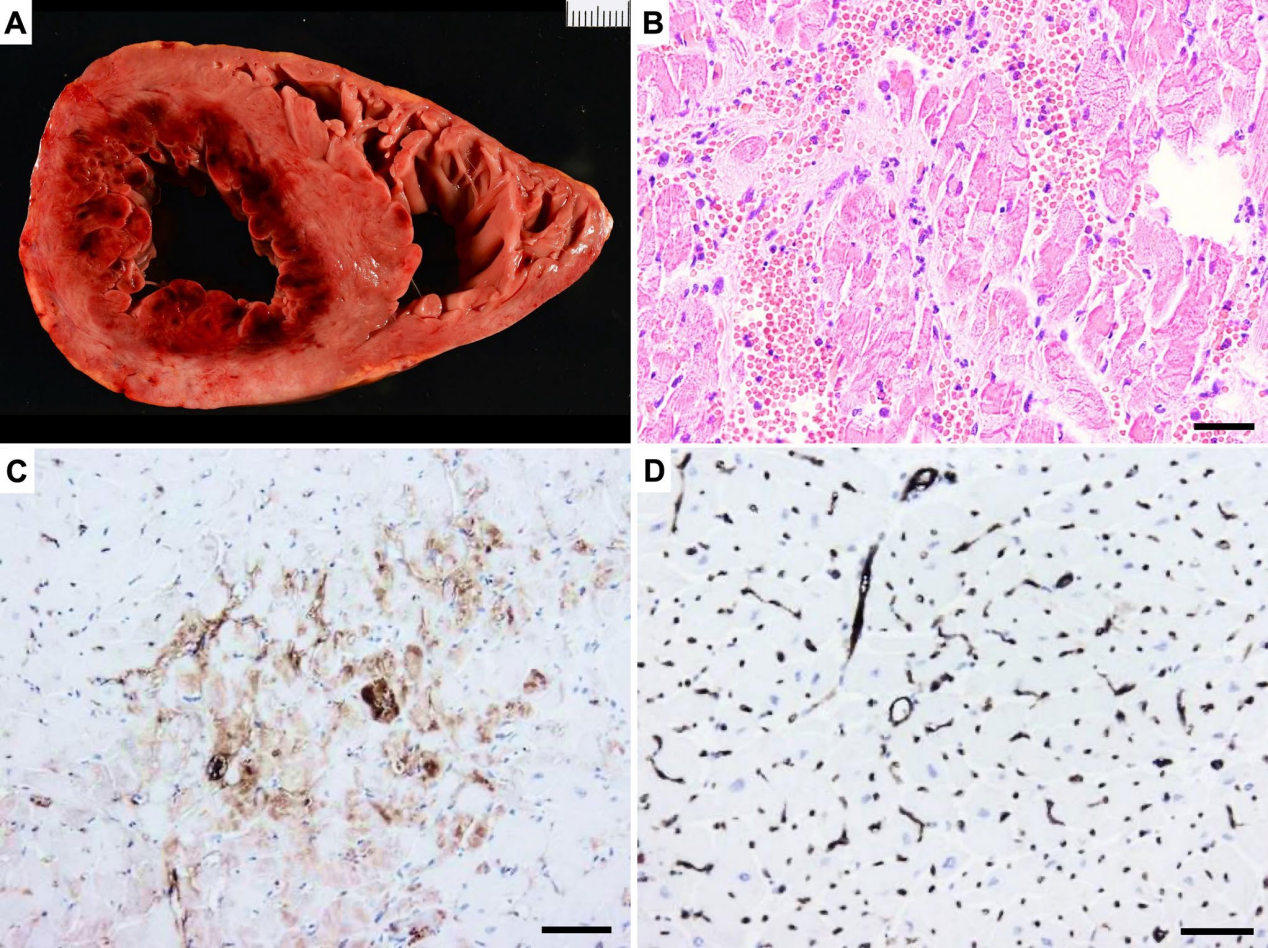
Ischemia & Reperfusion injury. (**A**) Macroscopy of fresh myocardial slice: circumferential subendocardial haemorrhage (‘red infarction’) in sudden cardiac death after prolonged resuscitative manoeuvres, H&E stain. (**B**) Microscopy: contraction band necrosis, polymorphous granulocytes infiltration, extravasation of erythrocytes, H&E stain, bar = 30 μm. (**C**) Microvascular destruction and leakage, anti-vWF immuno-stain, bar = 30 μm. (**D**) For comparison: microvasculature of normal myocardium, anti-vWF immuno-stain, bar = 30 μm

*Coronary intervention related injuries.* Restoration of the blood flow after a coronary occlusion, “coronary reperfusion”, may result in histopathological changes at autopsy that are morphologically clearly distinct from ischemic injury alone, and therefore designated as “reperfusion injury”. Late attempts to open the occluded artery, when large parts of the myocardium at risk are already significantly damaged, can lead to reperfusion injury, an exacerbation of cardiomyocytes injury (beyond ischemia alone) and microvascular dysfunction mainly due to the increasing production of reactive oxygen species (ROS) and inflammation. The pathological hall marks of reperfusion injury are: (1) widespread CBN (2) microvascular damage with extravasations of erythrocytes up to large hemorrhages, (3) microvascular occlusions (MVO) due to sludging and thrombosis of the vascular lumina. (4) abundant infiltration of PMN with widespread formation of neutrophilic extracellular traps (NETs). Together these phenomena may eventually result in a significant expansion of the infarcted area. At autopsy such reperfusion injuries may present grossly as large area of hemorrhage in the infarcted area (“red infarctions”), no reflow phenomena due to widespread microvascular dysfunction or obstruction, and even transmural or septal ruptures leading to cardiac tamponade (Fig. 3). In addition to these pathomorphological findings, it should be noted that occurrence of reperfusion immediately creates a situation of electrical instability in the reperfused area. Resulting arrhythmias are usually relatively benign (premature beats, accelerated idioventricular rhythms) but in some cases may also evolve to VF and therefore considered as a possible cause of sudden death immediately after reperfusion [17, 18].

### Myocardial injury due to non-ischemic causes

**Fig. 4:**
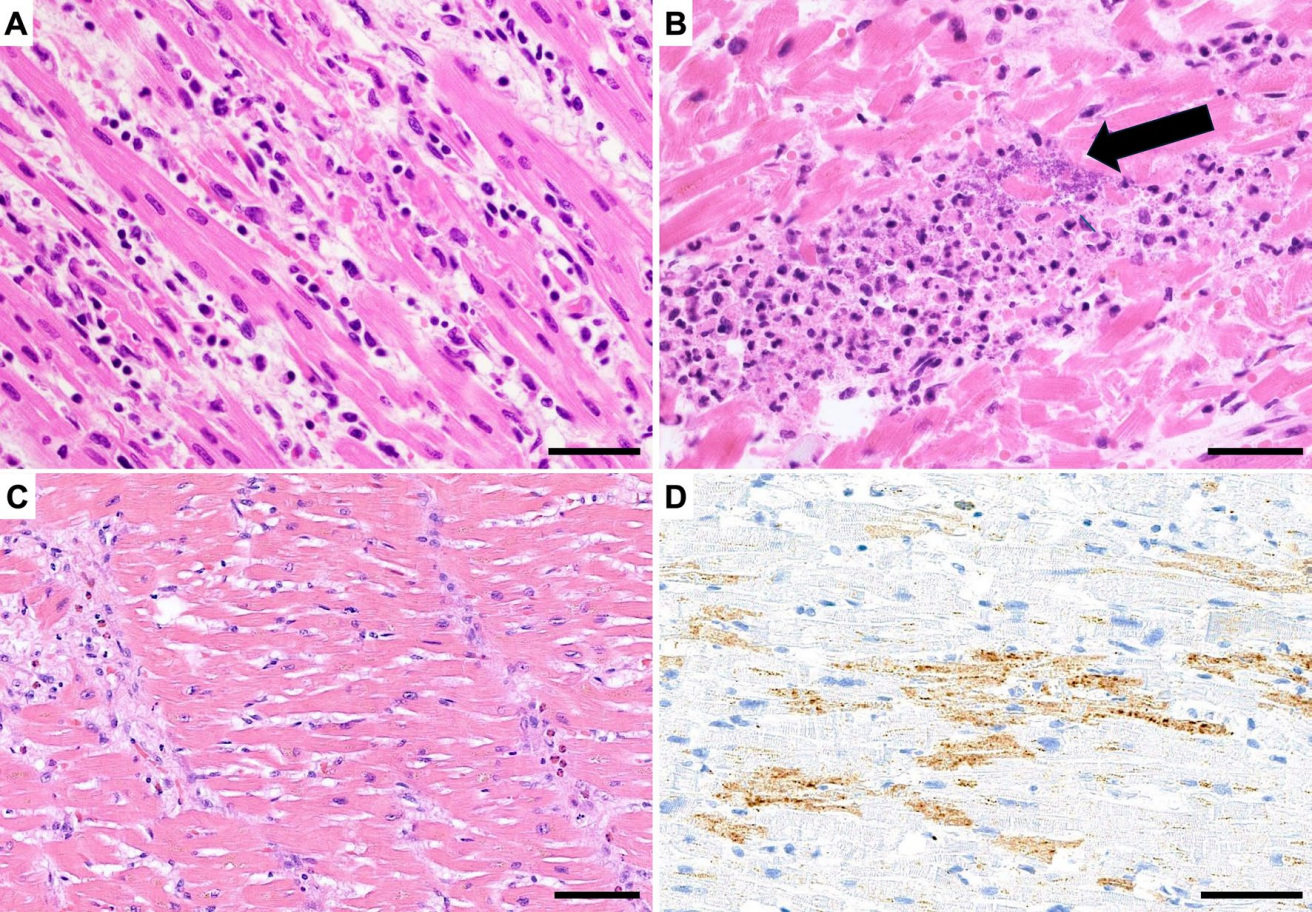
Non-ischemic myocardial injury: myocarditis. **A**) Acute stage myocarditis: myocyte injury associated with inflammatory cells, H&E stain, bar = 30 μm. **B**) Acute polymorphous myocarditis in sepsis (with evidence of bacterial colonies, arrow), H&E stain, bar = 30 μm. **C**) Acute non-lymphocytic (eosinophilic) myocarditis, H&E stain, bar = 50 μm. **D** Same case as in Fig. 4C: myocyte injury highlighted by immunostaining with C4d antibody, showing multifocal necrotic myocytes (brown), bar = 50 μm

**Fig. 5:**
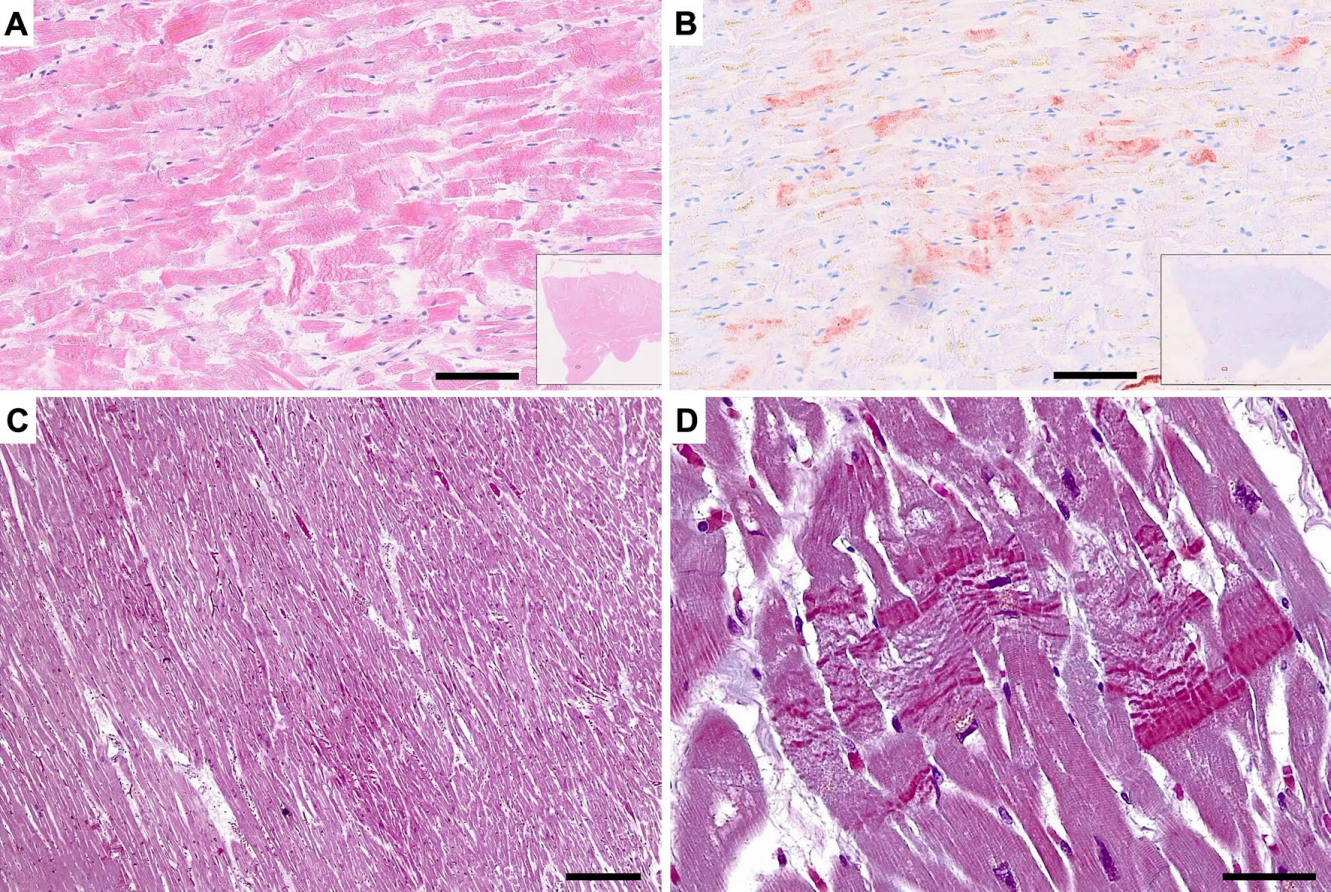
Extracardiac causes of non-ischemic myocardial injury. **A**-**B**) Cocaine abuse: contraction band necrosis and oedema, HE stains (**A**), immunostaining with C5b-9 antibody showing multifocal necrotic myocytes (**B**) bar = 50 μm. **C**-**D**) Myocyte injury in neurogenic myocardial injury; panel **D** is detail from same area as shown in panel **C** (H&E stain), bar = 200 μm in **C** and 30 μm in **D**

Many cardiac and systemic conditions can induce myocardial injuries and should be considered in the differential diagnosis of MI. They can be cardiac or extra-cardiac, and natural or non-natural (see Table 2) and such causes often co-exist and interact with a predisposition for cardiac ischemia. For example, during the early onset of COVID19 pandemic the presence of multiple types of myocardial injury, in the same patient, could be noticed at autopsy, which included interstitial myocardial macrophage infiltrations, lymphocytic myocarditis in a small fraction, and also other forms of myocardial injury such as right ventricular strain injury and thrombotic complications [19]. Relevant specific causes of myocardial injury are discussed in more detail below and myocardial and extra-cardiac examples are illustrated in Figs. 4 and 5.

#### Myocarditis

Myocarditis is broadly defined as an inflammatory disease of the heart. Inflammatory infiltration is a relatively common finding in forensic autopsies but not all inflammation should be considered as myocarditis. In the major textbooks of pathology “inflammation” is broadly defined as a “cell or tissue response to cell or injury” [20]. This implies that histologically, (irreversible) damage of the myocardium is a prerequisite for diagnosing myocarditis at autopsy. In autopsy studies focused on sudden cardiac death (SCD) incidences of myocarditis range from 0.3 to even 14.8% of cases [21]. Among patients with advanced cancers who were treated with immune checkpoint inhibitors, the incidence of myocarditis was reported as 1.14% [22]. Variations are related to patient populations involved (old versus young), and/or the extent of inflammatory disease in myocardium (minimal or borderline to fulminant myocarditis). There is still ongoing debate on how to diagnose myocarditis correctly and effectively at autopsy [23, 24]. Etiological backgrounds of myocarditis allow a subclassification of viral, immune, toxic and hypersensitivity forms of myocarditis, which may display distinct concomitant patterns of inflammatory cell infiltration (neutrophilic, lymphocytic, granulomatous, or with high admixture of eosinophils). In all these cases, signs of myocyte injury (in this setting often termed “ myocytolysis”) are noted, which in a recent survey was defined histologically as “vacuolization, fragmentation or disintegration of myocytes (groups or single cells) in the presence of inflammatory cells” [21]. These histological changes of myocardial injury can be diagnosed in H&E sections, but the application of immunohistochemical (IHC) stains specific for necrotic cells (as studied in experimental and human myocardial infarctions) can be helpful to confirm the diagnosis of any type of myocarditis [25].

#### Therapeutic and illicit drugs

Many drugs and illicit substances can damage the myocardium directly (toxic effects) and/or with hypersensitivity reactions and through the release of catecholamines [26]. Such effects are not necessarily dose-dependent and can vary between individuals. Interpretation of myocardial injury in a setting of (presumed) drug effects may require close collaboration with toxicologists.

In case of *toxic myocarditis*, cardiomyocyte injury is usually a direct toxic effect of drugs of abuse (amphetamines and related compounds, cocaine, opiates), anticancer drugs, catecholamines (dopamine, dobutamine, norepinephrine, epinephrine) and others. Histologically, toxic myocarditis is characterized by multiple foci of edema, myocytolysis inflammatory cells and fibrosis in various stages of development. The inflammatory infiltrate is usually composed of lymphocytes, plasma cells and PMN [27]. Direct toxic effects on the myocardium are generally assumed to be dose dependent. However, variation in response can be substantial between individuals with similar blood concentrations of the same substance.

*Hypersensitivity myocarditis* is considered a consequence of a delayed allergic reaction to the offending drug and therefore is not dose related. It is associated with numerous medications, including psychiatric medication (clozapine, lithium, tricyclic antidepressants), antibiotics (ampicillin, azithromycin, cephalosporins ciprofloxacin, isoniazid, penicillin, sulphonamides, tetracyclines), antiphlogistics (mesalamine, phenylbutazone) and others (dalimumab, colchicine, thiazide diuretics, methyldopa, dobutamine, lidocaine, metoprolol, phenytoin) [27, 28]. The histology of hypersensitivity myocarditis is characterised by focal or diffuse distribution pattern of inflammatory cells, where eosinophils predominate and with the same stage of development of myocardial lesions in all fields. Vasculitis can be noted in some cases.

*Cocaine* has a multitude of pathophysiological pathways through which it affects the cardiovascular system. The main acute effects of cocaine are due to sympathetic activation resulting in increased availability of stimulatory neurotransmitters and prothrombotic effects [29–31]. The sympathomimetic response includes increased myocardial oxygen demand (increased heart rate, increased contractility, and increased blood pressure). Oxygen supply is however limited by cocaine-induced vasoconstriction (or spasm) in both large and small vessels (small-vessel disease) due to α receptor stimulation, leading to myocyte ischemia. This risk is increased by the prothrombotic effects of cocaine (platelet activation and aggregation) which may induce intracoronary thrombosis as can be observed in large coronary arteries and/or small intracardiac arterioles. Cocaine is believed to be also directly cardiotoxic [29, 32, 33]. Histological changes in acute cocaine toxicity can, present with features of catecholamine-induced injury or ischemic injury. The findings may include scattered CBN and hypercontracted myocytes, or regions of ischemia in correspondence with coronary occlusion. Chronically there can be a myocarditis with scattered focal myocyte necrosis with or without CBN, or thesequelae of regional ischemia. Chronic findings can also include patchy nonregional fibrosis (thought to be due to small vessel disease), accelerated coronary artery atheroma, and microangiopathy in addition to myocardial inflammation [34].

*Ethanol.* Ethanol has direct toxic effects on the myocardium inducing an oxidative stress and mitochondrial dysfunction and, depending on genetic susceptibility, can result in alcoholic cardiomyopathy. There are no macroscopic or light microscopic features typical for alcoholic dilated cardiomyopathy [35–37].

*Anticancer medications.* Many chemotherapeutic agents induce myocyte injury by direct toxic effects. Anthracyclines (doxorubicin), alkylating drugs (cyclophosphamide, cisplatin), and taxanes (paclitaxel, docetaxel) are the most common chemotherapeutic medications linked to serious cardiac events. Myocardial injuries complicating anticancer therapies have been extensively studied, primarily focusing on the toxic effects of anti-tumor treatments on CMC, but also cardiac stromal cells and inflammatory cells T can be affected [38, 39]. The cardiotoxicity is due to multiple forms of cell death and myocardial dysfunction including necroptosis, pyroptosis, and apoptosis, autophagy, increased oxidative stress, and the inhibition of heart contractile function. Moreover, immunotherapy, which includes T cell therapy and immune checkpoint inhibitors as well as the radiation therapy also contribute [40, 41].

#### Trauma

Blunt trauma to the heart can be subdivided in contusion and incomplete or transmural laceration of the myocardium.

*Contusio cordis* has been reserved for cases of blunt chest trauma where there is cardiac bruising. Histologically, it is characterized by a well localized contused myocardium with hemorrhagic infiltrate, localized necrosis, and edema. It is not to be confused with *commotio cordis* which refers to cases of sudden cardiac (arrhythmic) death due to non-penetrating chest trauma without evidence of underlying myocardial disease or injury [42–44]. As mentioned above, these pathological findings often occur in combination with phenomena of reperfusion after cardiac arrest. Obviously, clinicopathological correlations are required in all these cases.

*Reperfusion injuries* can also occur in many situations of non-ischemic damage to the heart that are quite common in forensic practice. Blunt trauma to the heart, as in car accidents or other situations may result in traumatic irreversibly damaged, but nevertheless perfused myocardium (due to absence of coronary occlusion). The lesions are characterized at histological examination by *reperfusion phenomena* such as CBN and hemorrhage and can be in any location (including epicardial location, patchy pattern, involvement of the right ventricle). It can be difficult to distinguish such trauma-related lesions from ischemia-reperfusion as it occurs in myocardial infarction [15]. Reperfusion injury is a common finding following resuscitation and subsequent victim survival. These findings can be misinterpreted at autopsy as myocarditis or primary infarction [45].

*Cardiac ablation techniques*, usually applied in patients suffering from severe refractory arrhythmias, include a variety of procedures causing thermal injuries of which the most used are radiofrequency current and cryoballoon ablation [46]. The lesion consists of a central zone of coagulation type of necrosis surrounded with hemorrhage and inflammation. In the early stages a border zone of CBN and hemorrhage may be observed due to reperfusion. On the long term, these lesions evolve into scarring fibrosis, often with regular outlines (compact scars).

#### Extra cardiac causes of myocardial injury

*Catecholamine induced toxicity*,* Takotsubo syndrome and neuro-cardiogenic injuries.*

Catecholamines increase cardiac contractility, but exceptionally high blood levels can have adverse effects including cardiomyocyte damage. Such high levels can occur due to a variety of reasons, including acute emotional or physiological stress response, in cerebrovascular events, due to administration of adrenaline, or in the setting of a pheochromocytoma. A *catecholamine surge* can also be seen in violent deaths such as asphyxia deaths (hanging, drowning), in hypothermia, in adverse drug reactions, in long agony and in prolonged resuscitation in which catecholaminergic medications are administered [47, 48].

The injurious effects of catecholamines can be non-ischemic, direct on cardiomyocytes, and/or ischemic via inducing a decline in absolute myocardial blood flow or a rise in relative cardiac ischemia (i.e., stimulation of β-adrenergic receptors and higher energy demand) [49]. Direct effects are suggested to include calcium overload, oxidative stress, and mitochondrial dysfunction. The myocyte damage can present as CBN.

*Takotsubo syndrome (TTS*) is a clinical syndrome characterized (during life) by acute heart failure with transient regional myocardial wall motion abnormalities in the absence of significant coronary artery disease. In these patients, there is only a small elevation in troponin levels, not enough for the diagnosis of myocardial infarction. The syndrome is frequently preceded by a stressful emotional or physical trigger or can be secondary to an underlying disease such as pheochromocytoma and drugs inducing sympathetic overstimulation [50–52]. The pathophysiology is related mostly to the cardiotoxic effects of high levels of catecholamines, and the autonomic-limbic dysfunction might also play an important role in the pathophysiology of TTS [53]. The histopathology of TTS cannot be distinguished from (other) catecholamine-induced damage, neurocardiogenic damage or cocaine-induced damage. All these conditions are diagnosed through clinicopathological correlations [15]. Histologically, CBN and vacuolation of cardiomyocytes with widened interstitial space can be observed in the acute phase.

*Neurocardiogenic injury*. Sudden sympathetic stimulation, resulting in catecholamine surge may be due to several underlying causes, with intracranial processes (traumatic or nontraumatic, such as subarachnoid hemorrhage, intracranial hypertension and cerebral ischemia) being the most common. This is also known as “stroke-heart syndrome” [54–56]. The assumed pathophysiology has many similarities to TTS, with histologically similar types of myocardial injury.

*Sepsis* is a systemic inflammatory syndrome caused by infection that can develop into multiple organ dysfunction syndrome. Myocardial injury, as measured by release of cardiac troponins, is one of the most common complications and the main cause of death in septic patients. Several modes of cell death are implicated in septic injuries, including apoptosis, pyroptosis and autophagy [57]. Sepsis induced myocardial injury is still a challenging topic of (renewed) investigations and various mechanisms are considered, of which several are essential components of the host response to infection, mitochondrial damage, excessive inflammatory response with bursts of inflammatory cytokines, activation of coagulation, microvascular dysfunction, oxidative stress and catecholamine induced stress [5, 57]. Histologic lesions can be observed in most patients dying from septic shock. The distribution is widespread in all heart sections examined and not regional [58]. Histological parameters of stress induced cardiotoxicity were found in a series of 20 patients dying from septic shock, whose hearts revealed high rates of myocytolysis, CBN, mononuclear infiltrates interstitial edema and fibrosis. Some of them (30%) also had hemorrhages indicating microvascular damage and reperfusion [58]. It should be noted that 13 of these patients received catecholamine therapy and it is uncertain whether these pathological findings are related to endogenous or exogenous administered catecholamines, or whether ultimately mechanisms of ischemia and reperfusion may also be involved.

*Chronic kidney disease (CKD) / Renal failure.* The incidence of cardiovascular disease morbidity and mortality in CKD patients is higher than in the general population. The pathological pathways of myocardial injures in these patients involve coronary artery disease, volume overload, hypertension, diabetes mellitus, anemia, uremic toxins, renin–aldosterone–angiotensin system dysbalance, sympathetic nerve system activation, CKD-mineral bone disorder, malnutrition, inflammation, and oxidative stress [59, 60]. Because of this huge variety of pathologies, myocardial injury in the setting of CKD can be ischemic, non-ischemic or both.

### Distribution of myocardial injuries in the heart

The distribution of necrosis in the early stages and also the localization of scar tissue in the healing stages over the ventricular myocardium can help to determine the cause of myocardial injury.

Acute myocardial injury in the *subendocardium* usually corresponds with an ischemic origin. The pattern of damage may be regional or circumferential. Regional lesions occur because of acute critical flow reduction of an epicardial coronary artery. *Circumferential/multifocal myocardial* injury usually occurs because of an overall reduction in coronary perfusion pressure (e.g., hypovolemia or hypoxia) in subjects with severe multivessel coronary atherosclerosis. Irregular subendocardial hemorrhagic areas due to reperfusion injury may also be observed in subjects who die following cardiac arrest and cardiopulmonary resuscitation with a short time survival [15]. *A diffuse pattern of chronic subendocardial* damage can be observed in end-stage heart failure, usually associated with severe myocardial hypertrophy and dilatation. Persistent volume overload in these hearts with increased ventricular wall stress and consequent myocardial oxygen demand, leads to chronic ischemia. Colliquative myocytolysis, interstitial fibrosis and scarring are often observed. In case of coronary intramyocardial microvascular disease/dysfunction, a pattern of multiple *microscopic foci* of myocardial injury/scarring can be seen. In diffuse small vessel disease, these micro injuries may coalesce and be detectable even on gross cardiac analysis. When acute myocardial injuries or scars are *randomly distributed* within the myocardial thickness, non-ischemic damage should be suspected, such as myocarditis, toxic damage, and genetic cardiomyopathies. Myocardial injury is usually *patchy* and may involve only a small portion of the myocardium, with a preference for the subepicardial and midventricular layers. The distribution patters of myocardial injuries of any type (ischemic and non-ischemic) can be visualized in nitroblue tetrazolium (NBT) stains of fresh myocardial slices, represented by unstained areas at sites of myocardial necrosis in the heart (see below, ancillary techniques) [15].

## Diagnostic modalities at autopsy

In all forensic autopsies it is necessary to investigate the heart for the presence of myocardial injuries. To unravel the possible causes of myocardial injury, a detailed macroscopic and microscopic investigation of the heart should be applied as previously published. If available, it is important to screen all clinical information for presence of cardiac disease, including cardiac symptoms, (family) history of cardiac disease, resuscitation attempts, use of drugs (illicit / therapeutic) and finally ECG recordings. The next challenging step is to distinguish between ischemic injury (myocardial infarction) and non-ischemic injuries or a combination of both (as it can occur especially in the type 2 of MI).

In 2017 and 2023, respectively, two societies for cardiovascular pathology AECVP and SCVP published in depth investigation of the heart in victims of sudden cardiac death at autopsy [16, 61]. In addition, in 2020 AECVP published a review on postmortem diagnosis of the currently defined types of myocardial infarction [15] including its differential diagnosis. For a detailed investigation of the heart, we refer to the methodology as is outlined in these publications. Several ancillary methods to detect (early onset) myocardial injuries in autopsy material are applied in some institutions, partly in routine practice, and are still subject to further development and investigations. We are aware that these cannot yet be applied in every routine autopsy practice. Nevertheless, some of these have shown to increase reliably the diagnostic yield of evaluating myocardial injuries at autopsy and will therefore be discussed further in this chapter.

### Postmortem imaging (CT, MRI, angiography etc.)

In a clinical setting, the diagnosis of cardiac disease relies heavily on imaging techniques, especially echocardiography and magnetic resonance imaging (MRI). Imaging is also increasingly used as a diagnostic tool in a postmortem setting. This mostly pertains the use of full-body postmortem CT imaging (PMCT), but also includes the use of postmortem CT-angiography (PMCTA), postmortem magnetic resonance imaging (PMMR) and postmortem MRI-angiography (PMMRA) [62]. For ischemic injury, associated PMCT findings include coronary artery calcification and hemopericardium, but neither are diagnostic for myocardial infarction. Coronary calcifications are a ubiquitous finding in many adults, whilst proven cases of myocardial infarction can be entirely without coronary calcification [63]. Hemopericardium may be due to a ruptured necrotic infarct but can also be due to thoracic aorta dissection or perforation of the ventricle by rib fractures sustained in trauma or during cardiopulmonary resuscitation [64]. The coronary tree can be visualized with angiographic methods such as PMCTA and PMMRA, but its diagnostic value is limited by the possibility of postmortem clot and chronic vs. acute occlusion. PMCT has no added value for the diagnosis of myocardial injury on a cellular level. Better visualization is possible with PMMR, although postmortem change and temperature shifts pose substantial challenges compared with ante mortem imaging. Studies have shown the ability of PMMR to demonstrate cardiac edema, believed to be associated with cases of acute myocardial infarction [65–67]. However, cardiac edema is non-specific and may be a marker of any type of myocardial injury. Evaluating the significance of cardiac edema is hampered by the fact that it is not always visible histologically, and by the difficulty that it cannot differentiate between primary (disease-related) and secondary (agonal) cardiac damage.

### Nitroblue tetrazolium (NBT) stain of fresh transverse myocardial slice

The NBT stain is an enzymatic test that reacts only in areas of myocardium where lactate dehydrogenase is present. In fresh biventricular slices of the heart, unstained areas can be visualized, representing areas devoid of intracellular enzymes, corresponding with acute myocardial damage /necrosis. The test is reported to be positive already 3 h after onset of injuries, which means before the appearance of the histological alterations of myocyte necrosis. This method can be applied easily and provides excellent results but cannot discriminate between ischemic and non-ischemic types of injury. Care should be taken to avoid false positive results, which may occur, for example, in cases with a long postmortem interval (> 24 h) and resuscitation attempts with a short survival period [15].

### Immunohistochemistry

Immunohistochemical markers are extensively studied since years in the forensic context to detect an early myocardial infarction [68–71]. Of primary interest are the antibodies that recognize injured myocytes at the earliest stage of myocardial injury, and which, for daily practice, can be grouped in 3 categories: (1) antibodies recognizing proteins like fibrinogen, VWF or fibronectin, which accumulate in the in irreversibly injured cells; (2) antibodies reactive with several components of the complement system (especially C5B-9, C4D, C3); these can be expected to stain positively around the injured cells; (3) antibodies reactive with sarcolemmal proteins (actin, troponins, desmin) showing reduced or absent immunostaining at the site of injury. Proteins related to insudation or complement activation, stain positive before presence of PMN in the injured tissue, which can be observed around 6 h. In the complement cascade, C4d immunostaining seems very useful because it is a stable marker, which can be used on paraffin sections and it is widely applied in other situations of tissue injury (lung, kidney disease) [70]. IHC can be particularly helpful to delineate even very small foci of myocardial injuries or isolated dead cells in the heart (Fig. 1). The current list of IHC markers have been applied primarily to detect myocardial infarctions in (animal) experimental setting. They all stain positive at sites of necrotic cells, but they cannot discriminate between ischemic and non-ischemic forms of injury, such as for example in myocarditis (Fig. 4). It should be underlined also that the role of perimortem events, resuscitation and postmortem intervals on staining results are up to now insufficiently investigated to justify their routine application in forensic practice [68, 69]. Recent reviews showed reduction in staining in decomposed autolytic tissues [68]. C5-b9 is the only marker currently shown to be stable postmortem, supporting its use in cases with advances autolysis [69, 72].

In case of suspected myocarditis, immunohistochemistry, whenever available, can be applied on paraffin sections of trans mural myocardial slices, to evaluate not only the type of inflammatory cells, but also is useful to evaluate more accurately the inflammatory patterns (scattered, diffuse or multifocal granulomatous arrangement) as well as the extent and severity of the disease. In practice, application of only few antibodies, reactive with all leukocytes (CD45), T-cells (CD3) and macrophages (CD68) suffices in most cases. The full panel of antibodies, as recommended, includes antibodies reactive with T-lymphocytes (CD3), T-cell subsets (CD4, CD8), B-cells (CD20), macrophages (CD8) and plasma cells (CD138) [28, 73] (Fig. 4). Since the causes of myocarditis can be of natural or non-natural origin, additional toxicological, microbiological or IHC investigation should be carried out to differentiate between the two.

### Postmortem chemistry

Cardiac troponins (cTns) are the most sensitive and specific for diagnosing cardiomyocyte injury in clinical practice. Recently, cTns measurement was replaced by the high-sensitivity cardiac troponin T (hs-TnT) assay, able to detect MI within 3 h after the onset [1]. However, the increased levels of postmortem hs-TnT indicate presence of myocardial injury and are not specific for ischemia. Clinical evidence indicates that non-ischemic myocardial injury represents about 60% of cases of abnormal troponin concentrations. In autopsy practice, the postmortem cut-of values for serum and pericardial fluid, stability and changes associated with CPR are still the subject of debate [74–78]. It was suggested that low hs-TnT levels in pericardial fluid allow the reasonable exclusion of the heart damage [74], but a cut-off value as applied for in-vivo could not be established, probably because of postmortem alterations [76]. Most postmortem studies suggested that CPR has no effect on troponin levels [74–77]. It is generally accepted that postmortem measurement of cTn can be an auxiliary tool in the diagnosis of myocardial infarction/injuries.

## Evaluation of myocardial injuries at autopsy

**Fig. 6:**
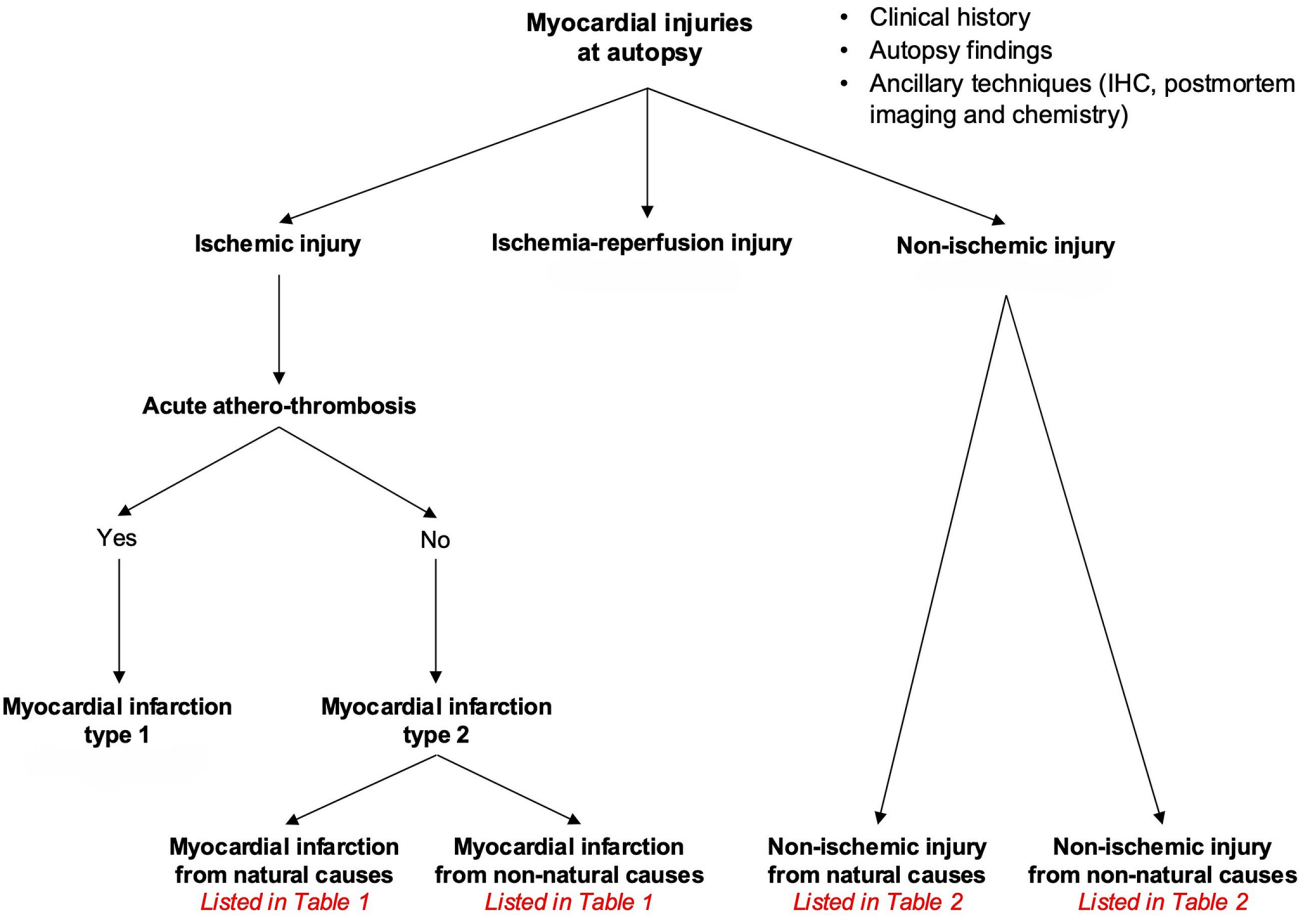
Flowchart to evaluate myocardial injuries at autopsy

The evaluation of myocardial injuries at autopsy is summarised in a flowchart presented in Fig. 6 and should be considered especially in the frequently confusing cases of myocardial injury where no thrombosed coronary plaque can be found. The first step is to *identify if there is any injury in the myocardium*. Noninvasive techniques as PMCT(A), PMMR(A), NBT stain of the fresh transverse myocardial slice and postmortem troponins can be applied, but they are not definitive in distinguishing between ischemic and non-ischemic injuries. Although some myocardial injuries can be suspected at macroscopic examination, histological examination is essential, and in selected cases the use of IHC and measurement of cardiac troponin may also be helpful. The second step is to *discriminate between ischemic and non-ischemic injuries.* All autopsy findings need to be interpreted with circumstantial data/ medical records if available and the results of ancillary analyses as toxicology, microbiology and postmortem chemistry [16]. At this step it is important to check the coronary artery pathology. Angiographic methods allow the detection of occlusion in one of the epicardial coronary arteries, but in all cases cardiac autopsy is required. At autopsy, the presence of coronary artery occlusion due to acute atherothrombosis indicates a type 1 myocardial infarction. In the absence of coronary artery thrombotic occlusion, also other causes of oxygen supply-demand imbalance could explain the ischemic injuries to the myocardium (myocardial infarction type 2, see Table 1). The causes can be natural (coronary, cardiac- non coronary or extracardiac, see Table 1) or non-natural (as a hemorrhage after a trauma, see Table 1). In the absence of potential ischemic causes of myocardial injuries, the next step should be to seek the non-ischemic origin. Here again, all available data and results should be evaluated. Non- ischemic injuries can be natural (as in sepsis, infectious myocarditis) or non-natural (such as toxic myocarditis, see Table 2 ).

## Conclusions

In clinical practice, the Fourth universal definition of distinct types of myocardial infarction, is presently considered gold standard [1]. In response, clinicopathological correlations have also recently been published in detail in the pathology literature [15, 79] emphasizing the importance to discriminate myocardial infarctions of any type from various other types of myocardial damage. The application of an approach to distinguish ischemic, non-ischemic and eventually combined forms such as ischemia and reperfusion injuries has proven to be particularly important (and challenging) in cases of sudden out-of-hospital cardiac arrest, and the ‘unwitnessed’ deaths at home in whom serious cardiac diseases can be found. This is even more important in forensic practice since differential diagnosis includes not only natural but also traumatic and iatrogenic causes of myocardial injury. In fact, myocardial necrosis, as the pathological substrate of various injury mechanisms, is frequently observed in postmortem (forensic) autopsy practice for both natural and non-natural deaths and should be interpreted with caution. The diagnosis can be straightforward, such as in a case with an acute thrombotic coronary occlusion or when cardiac tamponade due to cardiac rupture is found, but unraveling other causes of myocardial injury usually requires a detailed autopsy of the heart including histology. Moreover, assessment of the circumstances of death, resuscitation attempts, and all autopsy findings (especially of lungs, kidneys and brain) and data retrieved from biochemical, toxicological and microbiological investigations should help to orient the diagnosis in the first place towards an ischemic or non-ischemic origin of myocardial injury, and when possible, to the etiological cause of injury (as listed in the Tables 2 and 1). Therefore, a systematic approach is recommended as shown in the flowchart. Lastly, causality must be interpreted with care, in order not to misdiagnose agonal or resuscitation artifacts as the initial cause of the fatal event (death).

## Abbreviations

ACC: American College of Cardiology
ACS: Acute coronary syndrome
AHA: American Heart Association
AECVP: Association for European Cardiovascular Pathology
CABG: Coronary artery bypass graft
CBN: Contraction band necrosis
CKD: Chronic kidney disease
CPK: Creatin phosphokinase
CPR: Cardio-pulmonary resuscitation
ESC: European Society of Cardiology
IHC: Immunohistochemistry
LDH: Lactate dehydrogenase
MI: Myocardial infarction
MVO: Microvascular occlusions
NBT: Nitroblue tetrazolium stain
NETs: Neutrophilic extracellular traps
PMN: Polymorphonuclear leukocytes
PCI: Percutaneous coronary interventions
PMCT: Postmortem computed tomography
PMCTA: Postmortem computed tomography angiography
PMMR: Postmortem magnetic resonance
PMMRA: Postmortem magnetic resonance angiography
RAAS: Renin-angiotensin-aldosterone system
ROS: Reactive oxygen species
SCD: Sudden cardiac death
SCVP: Society for Cardiovascular Pathology
TAVI: Transcatheter aortic valve implantation
T1MI: Type 1 myocardial infarction
T2MI: Type 2 myocardial infarction
T3MI: Type 3 myocardial infarction
T4MI: Type 4 myocardial infarction
T5MI: Type 5 myocardial infarction
TTS: Takotsubo syndrome
WHF: World Heart Federation
VF: Ventricular fibrillation
VWF: Von Willebrand Factor
